# Efficacy of incentive spirometer and diaphragmatic breathing exercise on the alteration of arterial blood gas measures in patients after coronary artery bypass grafting: A randomized comparative trial

**DOI:** 10.1097/MD.0000000000045538

**Published:** 2025-10-31

**Authors:** Mohammad Abu Shaphe, Taimul Ali, Sanjay Kumar Pandey, Amir Iqbal, Faizan Z. Kashoo, Ramzi A. Alajam, Mohammad Hashim Khan, Ahmad H. Alghadir

**Affiliations:** a Department of Physical Therapy, College of Nursing and Health Sciences, Jazan University, Jazan, Saudi Arabia; b Department of Physical Therapy, Peerless Hospitex Hospital & Research Center, Kolkata, India; c Department of Cardiothoracic and Vascular Surgery, Batra Hospital & Medical Research Center, New Delhi, India; d Rehabilitation Research Chair, Department of Rehabilitation Sciences, College of Applied Medical Sciences, King Saud University, Riyadh, Saudi Arabia; e Department of Physical Therapy & Health Rehabilitation, College of Applied Medical Science, Majmaah University, Majmaah, Saudi Arabia; f Department of Diagnostic Radiology, College of Nursing and Health Sciences, Jazan University, Jazan, Saudi Arabia.

**Keywords:** ABG measures, CABG, chest physiotherapy, diaphragmatic breathing exercise, incentive spirometer

## Abstract

**Background:**

The incidence of lung-related issues after undergoing coronary artery bypass graft (CABG) surgery is notably significant. Initiating lung exercises soon after heart surgery plays a crucial role in averting these issues. The purpose of this research was to examine how the use of an incentive spirometer (IS) and diaphragmatic breathing exercises (DBE) impacts changes in arterial blood gas (ABG) levels.

**Methods:**

The study was based on a 2-arm, parallel-group, randomized comparative design. In this study, 30 individuals who had CABG surgery were selected based on specific criteria and randomly divided into the IS group and the DBE group. Each group received targeted chest physiotherapy; the IS Group had an IS, and the DBE Group had DBE. Key ABG parameters, such as blood pH, partial pressure of arterial oxygen (PaO_2_), and partial pressure of arterial carbon dioxide (PaCO_2_), were evaluated using an ABG analyzer. Measurements were taken initially on the first day after extubation and then again on the second day post-extubation. All statistical analyses were conducted with a maintained significance threshold of 95%.

**Results:**

The results showed no significant differences between-group and within-group comparisons for PH, PaO_2_, and PaCO_2_ outcomes. However, the IS group patients showed a trend in the increase in PaO_2_ levels and a decrease in PaCO_2_ levels compared to those in the DBE group.

**Conclusion:**

Despite not revealing statistically significant differences in the alteration of ABG measures between groups, the current study observed minimal improvements in ABG measures in the IS Group compared to the DBE Group. Therefore, this study suggests that, in addition to conventional chest physiotherapy, using an IS might be superior to DBE in altering ABG measures in patients with post-CABG. Nonetheless, we may use both intervention and conventional chest physiotherapy postoperatively as a prophylactic regime.

## 1. Introduction

Coronary artery bypass grafting (CABG) is a widely practiced treatment method globally. Data indicates that annually, over 1 million CABG surgeries are carried out. Pulmonary complications are one of the most common consequences post-CABG. The incidence of pulmonary complications is between 30% and 60%. Chest pain, generalized weakness, reduced chest mobility, ciliary paralysis, fluid retention, impaired oxygenation, and inconsistencies in gas exchange are primarily associated factors in pulmonary complications, such as hypoxemia, pneumonitis, and atelectasis following CABG existing in patients due to the close relationship between heart and pulmonary system.^[1,2]^ The pulmonary complications following CABG might increase the risk of morbidity and mortality, hospital costs, and the duration of hospital stay.^[1–3]^ Hypoxemia, pneumonitis, and atelectasis have long been recognized as a major cause of morbidity in the postsurgical cardiac patient. It has been estimated that the incidence of these complications is 2.5% to 3% for all operations.^[3]^

Despite meticulous fluid and respiratory management, the occurrence of atelectasis following heart surgery with cardiopulmonary bypass remains high, between 80% and 84%.^[4]^ The primary respiratory issue in patients after surgery is atelectasis, which leads to hypoxemia and consequently alters arterial blood gas (ABG) levels. Atelectasis is associated with several conditions: an increased alveolar–arterial O_2_ difference due to elevated right-to-left intrapulmonary shunting, a reduced ventilation–perfusion ratio, a lowered functional residual capacity, and diminished compliance.^[4]^ As a preventive measure against these lung-related complications post-surgery, chest physiotherapy is commonly employed after significant abdominal and cardiothoracic surgeries.^[3,4]^

Literature shows that identifying postoperative complications is paramount to preventing pulmonary complications.^[1]^ There are several types of physiotherapy management available to prevent pulmonary complications.^[5]^ The techniques include deep breathing training, intermittent positive pressure breathing, DEVICES, positive airway pressure ventilation, and early ambulation.^[1,2]^ Following CABG, physical therapists focus on breathing exercises emphasizing the deep inspiration technique with the use of IS devices to clear bronchial secretion and early mobilization to improve respiration and prevent the risk of lung complications, such as pneumonitis and atelectasis.^[3–5]^ The primary role of diaphragmatic breathing exercise (DBE) and incentive spirometer (IS) is to improve ventilation and reduce the ventilation-perfusion mismatch, the usual mechanism of ventilation-perfusion mismatch and decreased O_2_ saturation may be attributed to the inability to exert sufficient inspiratory effort to reach a high lung volume secondary to pain and muscle weakness.^[4,5]^ The use of the DEVICE was first described in 1970 in order to reduce postoperative pulmonary complications. The main function of this device is to encourage patients to achieve maximal expansion of the chest during inspiration by providing visual feedback.^[6,7]^

The IS maneuver provides a means of maximally inflating the lung by generating a native intrapleural pressure. This maneuver is a voluntary action of the patient who must take large breaths resembling the sign or yawn mechanism. In normal health, these maneuvers are performed many times each hour and it has been shown that the absence of such deep breathing can lead to a progressive alveolar collapse in as little as 1 hour.^[8,9]^

Currently, opinions vary regarding the efficacy of the 2 main breathing exercises (IS and DBE).^[10–12]^ Therefore, a systematic examination of their effectiveness is undertaken in this research. The goal of this study is to assess the impact of IS and DBE, alongside regular chest physiotherapy, on modifying ABG levels in patients after CABG surgery. The hypothesis of this research is that a notable distinction will be observed in the influence of IS and DBE, when combined with standard chest physiotherapy, on the alteration of ABG in patients who have undergone CABG.

## 2. Materials and methods

### 2.1. Study design

This research adopted a 2-arm, parallel-group, randomized comparative trial design. It included patients who had undergone CABG surgery. The study used an online website, randomization.com, to allocate the participants to either of the groups. This method ensures that the assignment of participants to different groups is random, which is crucial for maintaining the integrity and validity of the study.

### 2.2. Sample size

The computer software G*Power 3.1.9.4 was used to calculate the effective sample size for this study. A computer a priori *t*-test for 2 dependent means was analyzed, keeping the significance level at 95% (2-tailed), power at 80%, the error probability of 5%, and the effect size of 0.52. A total sample of 32 patients (n = 16/group) was estimated to ensure enough power for the study sample. Assuming a 30% sample attrition, 42 participants were estimated. Outcome score of PaO_2_ (mean difference = 4.57; standard deviation difference = 8.85) in a pilot study were used for estimating the sample size of this study.

### 2.3. Study settings

The patients with post-CABG were directly approached for the study’s screening and recruitment from the hospital’s postsurgical intensive care unit, cardiothoracic and vascular surgery department. The study data collection was completed in about 4 months, from April 15, 2019, to August 25, 2019.

### 2.4. Participants

A total of 30 patients with post-CABG were recruited for this study based on inclusion and exclusion criteria and then randomly allocated into one of 2 groups (IS Group and DBE Group) using an online website randomization.com <www.randomization.com>. The patient with post-CABG, aged between 40 and 60 years, had forced expiratory volume in 1 second < 70% of the predicted value, showed forced expiratory volume in 1 second/ Forced vital capacity ratio > 0.8, weight equal or exceeded the ideal body weight by <20%, and had respiratory extubated post-CABG, were included in the study. However, patients were excluded from the study if they had one of the following conditions: aged other than 40 to 60 years, weight equal or exceeded the ideal body weight by more than 20%, had respiratory intubation post-CABG, history of chronic obstructive pulmonary disease/thoracic surgery including CABG, thoracic anomalies/unstable angina, developed hemodynamic complication, or non-cooperative/neurological debilitated patient.

### 2.5. Outcome measures

The outcome measure, including the PH of blood, PaO_2_, and PaCO_2_, was assessed by an ABG analyzer. The instruments IS and ABG analyzers were used in this study.

### 2.6. Procedure

**Figure 1.**
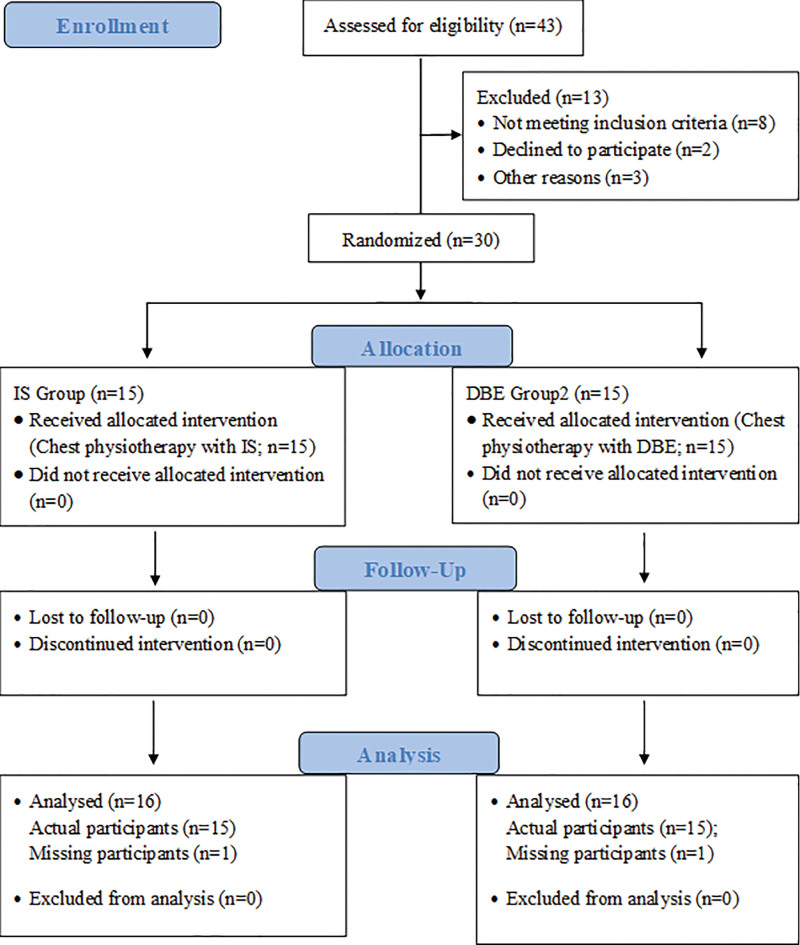
A CONSORT (2010) flow chart is depicting the study procedures, including enrollment, allocation, follow-up, and analysis.

Before the CABG surgery, an assistant physical therapist who was blinded to the study completed the assessment part, including the plan of CABG surgery (performed via median sternotomy), and submitted it to a specialist physiotherapist. Specialist physiotherapists identified 30 potential candidates for the study after preliminary screening based on inclusion and exclusion criteria, randomly allocated to both IS and DBE groups. Participants were easily available, could spend time, and showed interest in participating in the study. An online website, randomization.com (www.randomization.com), was applied to allocate the participants into one of the study groups (n = 15/group). To reduce the risk of bias in this research study, a peer researcher reviewed it thoroughly and checked the equipment for standardized calibration; in addition, data was recorded accurately and maintained carefully. Furthermore, he/she explained to patients the need for early mobilization post-CABG surgery. He/she taught them how to clear excess bronchial secretions from the lungs, including huffing (forced expiration with the glottic open), coughing, and active exercise of the upper and lower extremities. To minimize pain at the incisional site, a chest binder was used to support the incision, and patients were taught to do huffing and cough with the sterna supported with their hands. In addition, the patients in both groups were given a rationale for using a volume-oriented DEVICE (IS Group) and for DBE (DBE Group). They were also treated according to their stipulated protocols once extubated on postoperative day 1. With the permission of a resident doctor, confirming the stable and safe condition, and considering the oxygen supplementation by nasal prong post-extubation, routine chest physiotherapy was performed on both groups of patients. Baseline measurements for the outcomes were taken on post-extubation day 1, before starting the intervention. Chest physiotherapy was performed 3 hours post-extubation to avoid post-extubation clinical manifestations. Each group received 3 sessions daily in the morning between 8 am and 9 am, afternoons between 1 and 2 pm, and evening between 6 and 7 pm. Each session lasted for 30 minutes. Each participant received 3 sets of chest physiotherapy with 2 minutes rest intervals. In addition to the chest physiotherapy, the patients in the DBE group were taught to perform 3-5 DBE in each set with 2 minutes of rest intervals for 3 sets each session. The patients from IS Group were instructed to use an IS device for 3 to 5 consecutive breathings for 3 sets with an interval of 2 minutes of quiet breathing each session in the sitting or semi lying position. However, the patients in the DBE Group were instructed to perform the DBE in addition to conventional chest physiotherapy, including postural drainage (percussion and vibration in optimal position per compartment/chamber of lungs) and normal inhalation and exhalation of breathing in the sitting or semi lying position. Additionally, the patients in both groups were instructed to practice self-treatment exercise preoperatively, one day before surgery, one group was taught how to perform DBE, and the other group was taught how to use IS thrice daily so that the subject could easily understand the correct pattern and technique and be supposed to be performed the same during each waking hour until the end of postoperative day 3, including huffing, coughing with sternal support, active exercise for the upper and lower extremities, incentive spirometry (at least 10 times in IS Group) and DBE (at least 10 times in DBE Group). The duration of the treatment session lasted an average of 10 to 15 minutes for patients in each group, excluding the time of conventional chest physiotherapy. The postintervention data were collected until the participants stayed for 2 days, i.e., day 2nd and 3rd postintervention. A CONSORT (2010) flow chart depicts study procedures such as enrollment, allocation, follow-up, and analysis in Figure 1.

### 2.7. Interventions

**Figure 2.**
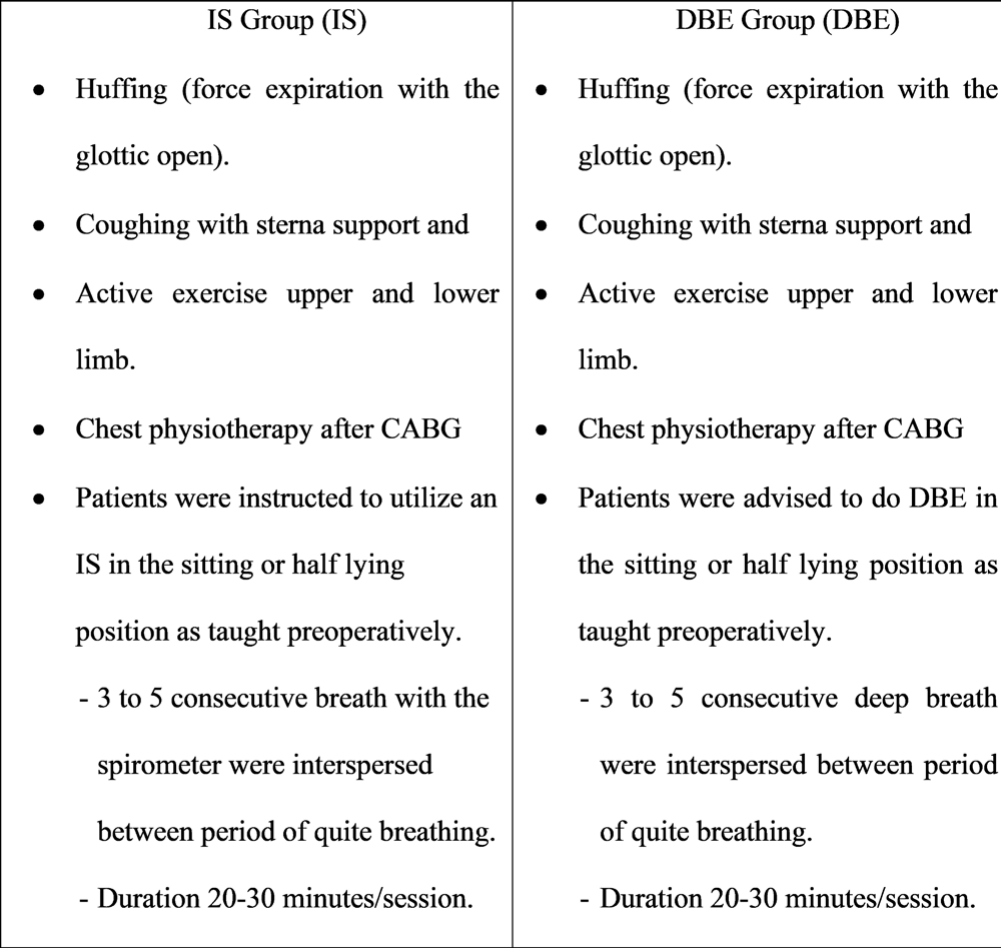
Intervention protocols for the IS and DBE groups. DBE = diaphragmatic breathing exercise, IS = incentive spirometer.

Besides the IS (IS group) and DBEs (DBE group), patients in both groups were introduced to practice self-treatment exercises preoperatively and were asked to be performed successfully postoperatively, as described in Figure 2.^[3,13]^

### 2.8. Ethical consideration

The current study was approved by the Research Ethics Sub-Committee at King Saud University (file ID: RRC-2019-13; dated 08-04-2019). A trial registration was sought for the study from the ClinicalTrials.gov Protocol Registration and Results System (identifier ID: NCT05684679; dated January 13, 2023). The current study followed research ethics guidelines and maintained human rights while conducting the research. The study was carried out in accordance with the Declaration of Helsinki (2010). A signed informed consent form was obtained from each patient before the start of this study (at pre-CABG).

### 2.9. Data analysis

Data were analyzed using the software package SPSS V.16 and STATA 8.0. The total sample size of the study was limited to 30 instead of 32. However, data analysis was done for 32 participants by adding the average values of demographic and dependent variables of 30 participants into the missing 2 participants’ data. A Chi-square test was performed to compare the categorical variables between groups at baseline. Repeated measurement ANOVA was to perform the effects of either IS or DBE delivered with chest physiotherapy on the values of ABG within each group and between the groups across different time points. A Post Hoc test using the LSD method was run to compare the differences between groups (IS and DBE) for all outcome scores at each time point. All *P*-values referred to 2 side tests, and statistical significance was set below .05.

## 3. Results

Thirty out of 43 patients with post-CABG were assessed for the study’s eligibility. Thirteen out of 43 patients were excluded from the study due to various reasons, such as not meeting the inclusion criteria (n = 8), declining to participate (n = 2), and other reasons (n = 3). Demographic details for homogeneously distributed data and baseline scores for the outcomes of the PH of blood PaO_2_ and PaCO_2_ were described as given in Table 1. The statistical differences in the results were reported within each group for all outcomes across different time points and between the groups at different times.

**Table 1:** Descriptive characteristics of the patients with post-CABG (N = 30).

Sl.	Variables		IS group	DBE group
01.	Mean age (yr)		50.33	48.79
02.	Weight (kg)		78.15	83.76
03.	CPB time (min)		98.16	107.21
04.	Type and No. of graft	CABG × 1	3	2
		CABG × 2	6	10
		CABG × 3	6	3
		AVR	1	0
		MVR	0	1
05.	Graft used	1	3	2
		1,2	6	10
		1,2,3	1	1
		1,2,4	5	1
		1,3,4	0	1

Graft used: [1] Lima; [2] Saphenous; [3] Left Radial; [4] Right Radial.

AVR = aortic valve replacement, CABG = coronary artery bypass graft, CPB = cardiopulmonary bypass, DBE = diaphragmatic breathing exercise, IS = incentive spirometer, MVR = mitral valve replacement.

### 3.1. Within-group comparison

The within-group comparison revealed an insignificant improvement when comparing the baseline scores for the outcomes PH of blood PaO_2_ and PaCO_2_ on day 1st and 2nd postintervention scores within groups (IS and DBE) as described in Table 2.

**Table 2:** Comparing the scores for the outcomes PH of blood (PH), arterial partial pressure of oxygen (PaO_2_), partial pressure of carbon dioxide (PaCO_2_) within IS and DBE Groups (n = 15/group) using repeated-measures ANOVA (95% CI).

Variables		Scores over different time duration (mean ± SD)			Repeated-measure ANOVA	
Outcomes	Groups	Pre-intervention	Post intervention			
		Baseline	Day 1st	Day 2nd	*F*-value	*P*-value
PH	IS group	7.44 ± 0.04	7.43 ± 0.03	7.44 ± 0.02	0.19	.90
	DBE group	7.43 ± 0.04	7.44 ± 0.04	7.43 ± 0.04	0.60	.62
PaO_2_	IS group	98.34 ± 34.88	106 ± 38.52	107 ± 42.12	0.86	.46
	DBE group	102.57 ± 34.02	113.38 ± 39.19	105.05 ± 42.85	0.44	.72
PCO_2_	IS group	34.29 ± 5.51	35.29 ± 5.93	35.18 ± 3.47	0.25	.86
	DBE group	33.73 ± 4.42	34.01 ± 5.11	34.27 ± 3.25	0.12	.94

Data are presented in mean ± standard deviation (SD). The level of significance set at *P*-value < .05.

DBE = diaphragmatic breathing exercise, IS = incentive spirometer, PaO_2_ = partial pressure of arterial oxygen, PCO_2_ = partial pressure of arterial carbon dioxide.

### 3.2. Between-group comparison

Similarly, the between-group analysis revealed an insignificant difference (*P* > .05) when comparing the scores for the outcome PH of blood, PaO_2_, and PaCO_2_ between IS Group and DBE Group at baseline and day 1st and 2nd postintervention, as described in Table 3.

**Table 3:** Pair-wise comparison of the scores for the outcomes of PH of blood, arterial PaO_2_, and PaCO_2_ between IS Group (n = 15) and DBE Group (n = 15) at different time durations post-operation, using a post hoc test (multiple comparison LSD).

Outcomes	Groups	Scores at different time points (∆MD±∆SD)			Post hoc test (LSD)	
		Pre-intervention	Post-intervention			
		Baseline	Day 1st	Day 2nd	*F*-value	*P*-value
PH	IS vs DBE	0.01 ± 0.004	−0.01 ± −0.008	0.01 ± −0.021	0.05	.824
PaO_2_	IS vs DBE	−4.23 ± 0.86	−7.38 ± −0.66	1.95 ± −0.73	0.44	.72
PaCO_2_	IS vs DBE	0.56 ± −1.09	1.28 ± 0.82	0.91 ± 0.22	0.42	.52

Data are presented in mean ± standard deviation (SD). The level of significance set at *P*-value < .05.

DBE = diaphragmatic breathing exercises, IS = incentive spirometer, ∆MD = mean differences, n = number of patients in the group, PaO_2_ = partial pressure of arterial oxygen, PCO_2_ = partial pressure of arterial carbon dioxide, ∆SD = differences in standard deviation.

## 4. Discussion

This study aimed to assess the effectiveness of the use of an incentive spirometry device versus DBE with ABG alteration in patients who underwent CABG. The results of the study showed no significance differences in ABG values between groups. The incentive spirometry group showed a trend for an increase in the level of PaO_2_ and a decrease in the level of PaCO_2_ compared to the DBE group. However, the level of the PH values showed no difference in the incentive spirometry group compared to the DBE group.

Following CABG, the chance of developing atelectasis is almost 80% to 84% despite taking normal respiratory care.^[4,14–16]^ As we have seen in our findings, on the second postoperative day, all patients developed atelectasis in the basal part of the lung, so it is understandable that in the first 2 days, there was a minimum amount of atelectasis which was developed in all participants. The within-group comparison showed a trend for an increase in PaO_2_ and a decrease in PaCO_2_ in the IS and DBE groups. The results of our study revealed a higher increase in the level of oxygenation of the IS group compared to the DBE group. The effect of atelectasis was reduced from the second postoperative day in both groups. In the clinical setting, exercises were repeated every hour and every day, but it is very unlikely that an increase in the repetition of exercises might have a continuous substantial effect; however, this field requires further observation.^[17–19]^ Our findings are in parallel with a previous study conducted by Ahamdereza Yazdannik et al who reported almost similar effects of incentive spirometry on ABG values when compared to normal breathing exercise. They concluded that incentive spirometry might be superior compared to normal breathing exercises.^[1,20–23]^

In the current study, we examined using an incentive spirometry device while performing DBE because it provides visual feedback and is easily available at the hospital where the study was conducted. The focus was to facilitate deep breathing by providing visual feedback to the patients while performing maximal slow inspiration. The patients in the DBE group were informed to perform DBE once per hour throughout the day. The frequency of exercise (3 sets of 10 breaths) was determined based on the ordinary routine at the clinics for both groups. Currently, it is unknown whether increasing the intensity and frequency of the exercise is possible to be more effective. All patients reported that breathing exercises were easy to perform, and most encountered some subjective benefits of the exercises, such as patients feeling relaxed, reduced stress, etc.

The incentive spirometry device is one of the common pieces of equipment used during cardiac rehabilitation. It helps increase the level of PaO_2_, rapidly binding oxygen molecules to hemoglobin molecules. Thus, improving the oxygen saturation percentage in the oxygen dissociation curve. The more the oxygen molecules combined, the supply of oxygen to the tissue would improve, and tissue healing would be faster. It will reduce the staying in the hospital.^[10,24]^ A systematic review study showed physiological evidence that using IS devices may be appropriate for increasing lung expansion after major thoracic surgery. Therefore, the literature directly supports our study and provides information on the requirement of incentive spirometry^[11,25]^ According to Moradyan et al., incentive spirometry combined with DBE has shown significant improvement in the level of PaO_2_ and arterial oxygen saturation (SaO_2_) in post-CABG patients. Although the result of the study cannot explain which method is more effective compared to others for improving oxygenation, it cannot be denied that these exercises cause improvement in oxygenation and gas exchange in view of pulmonary function.^[12,26–28]^ The literature indirectly supports our findings. On the other hand, the effectiveness of incentive spirometry is still controversial. Incentive spirometry does not provide significant changes in arterial blood gas measures.^[13,29–31]^

In this research, we found no significant distinction in effectiveness between the IS and the DBE. This suggests that a basic, cost-effective tool could be just as efficient as more sophisticated and costly options, such as intermittent positive pressure breathing or flutter devices. Simply placing a device near a patient’s bed is insufficient to motivate regular usage. Therefore, it’s advised that either IS or DBE should be paired with consistent encouragement and repeated guidance every day after surgery.

Despite these benefits, a few shortcomings are observed in this study. First, the study was limited to male patients only by chance and a specific age group between 40 and 60. Second, the sample size of the study was relatively small. Third, we did not evaluate the incentive spirometer’s frequency and long-term effect. We did not know that increasing the repetition does help to improve the ABG values. According to Brage’s statement, the improvement in blood oxygenation following breathing exercises is a short-term effect, so long-term improvement in blood oxygenation requires increasing the repetition and duration of breathing exercise.^[14,28]^ In addition, this study collected only 2-day postintervention data instead of the complete duration of hospitalization. Therefore, a multi-centric study is required to examine the long-term effect of the incentive spirometer DBE, to conduct more than one medical center which is well equipped and ultra-modern machines are available. Effect of increasing repetition number, optimizing the dose of exercise on ABG values in a wider age group and a larger sample of CABG patients until the complete duration of hospitalization. Furthermore, instead of comparing DBE versus IS, it is advised to investigate the effect of breathing patterns and ventilatory strategies on any ABG alteration post-CABG.

## 5. Conclusions

Our research did not find significant statistical differences in the alteration of ABG parameters between the groups. However, we noticed slight enhancements in ABG readings in IS group compared to the DBE group. The incentive spirometer offers several benefits, including visual biofeedback for patients, which DBE lacks. It is also readily accessible and user-friendly, aiding in improving lung compliance without needing therapist assistance once patients are proficient in its use. Consequently, our study proposes that incorporating an IS along with standard chest physiotherapy may be more beneficial than DBE alone for post- CABG patients. Yet, employing both IS and DBE alongside conventional chest physiotherapy postoperatively could serve as an effective preventive approach. The use of IS notably enhances arterial blood gas outcomes (PaO_2_, SaO_2_, & PaCO_2_) following CABG.^[1]^

## Acknowledgments

The authors extend their appreciation to the Deanship of Scientific Research, King Saud University for funding through Vice Deanship of Scientific Research Chairs; Rehabilitation Research Chair.

## Author contributions

**Conceptualization:** Mohammad Abu Shaphe, Taimul Ali, Sanjay Kumar Pandey, Ramzi A. Alajam, Amir Iqbal, Ahmad H. Alghadir.

**Formal analysis:** Amir Iqbal, Faizan Z. Kashoo, Mohammad Hashim Khan, Ahmad H. Alghadir.

**Funding acquisition:** Ahmad H. Alghadir.

**Methodology:** Taimul Ali, Sanjay Kumar Pandey, Faizan Z. Kashoo, Ramzi A. Alajam.

**Writing – original draft:** Mohammad Abu Shaphe, Taimul Ali, Sanjay Kumar Pandey, Amir Iqbal, Ramzi A. Alajam, Mohammad Hashim Khan.

**Writing – review & editing:** Mohammad Abu Shaphe, Sanjay Kumar Pandey, Amir Iqbal, Faizan Z. Kashoo, Ramzi A. Alajam, Mohammad Hashim Khan, Ahmad H. Alghadir.
